# Insulinoma Causing Liver Metastases 15 Years after Initial Surgery, Accompanied by Glomerulonephritis

**DOI:** 10.1155/2012/168671

**Published:** 2012-12-03

**Authors:** A. Janez

**Affiliations:** Department of Endocrinology, Diabetes and Metabolic Diseases, University Medical Center Ljubljana, Zaloska 7, 1000 Ljubljana, Slovenia

## Abstract

Insulinoma is a rare pancreatic endocrine tumor that is typically sporadic, solitary, and less than 2 cm in diameter. Ninety percent of all insulinomas are benign. Up to 10 percent are malignant and are usually larger in size. We report a case of an unusual, not yet described association of two diseases: a malignant pancreatic insulinoma recurred as a multiple liver metastasis 15 years after the initial complete enucleation of a primary tumor with a histomorphological fairly benign outlook, accompanied by ANCA positive crescentic glomerulonephritis.

## 1. Introduction

Insulinoma is the most common endocrine tumor arising from beta cells of the pancreas. It has an estimated incidence of 1–4 cases/million/year, based on population study [1]. The median age of patients at presentation is approximately 47 years and is slightly more prevalent among woman [2, 3]. About 90% of insulinomas are benign, solitary tumors, evenly distributed over the pancreas, and measuring less than 2 cm [2–4]. Symptoms are usually related to the excessive insulin secretion and can be divided into either neuroglycopenic with episodes of visual changes, incoherence, seizures and even coma, or adrenergic caused by counter-regulatory catecholamine excess, such as weakness, palpitation, sweating, and pallor [1]. Up to 10 percent of all insulinomas behave biologically as malignant tumors. Metastases from these tumors to the liver and lymph nodes are usually hypervascular [5, 6]. Exact localization of the tumor by exclusion of metastases is the main goal of preoperative evaluation. The differential diagnosis between malignant and benign insulinoma is usually very difficult and is based on intraoperative evidence such as metastases in the liver, regional nodes, or local invasion, whereas in some patients the metastases are frequently found along with the recurrence of a hypoglycemic episode [3, 6, 7]. We had a rare opportunity to study a case of insulinoma with an unusual course of the disease.

## 2. Case Report

**Figure 1:**
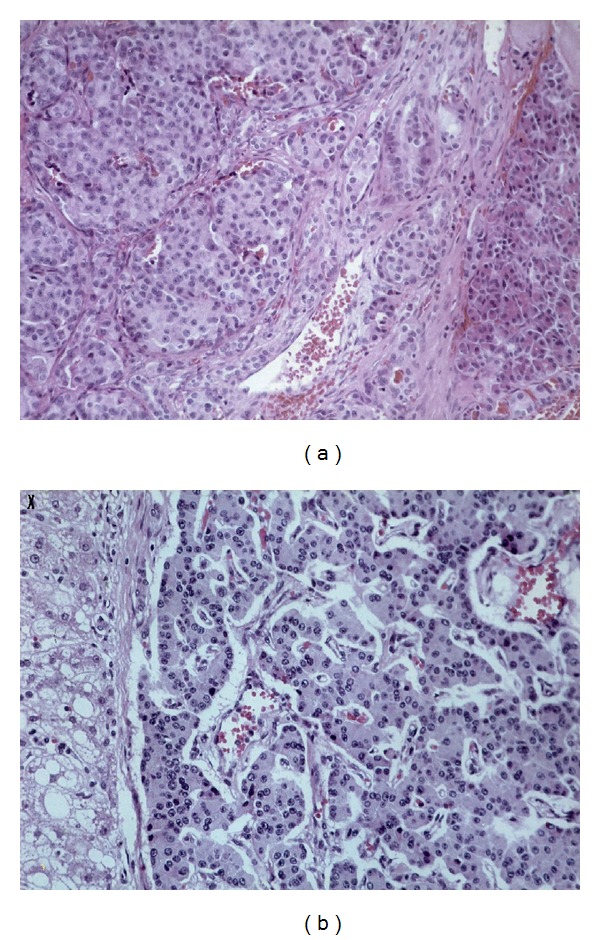
(a) Microscopic features of the excised insulinoma. Histomorphologically, a solid pattern was predominated. Moderate sized tumour cells formed solid cellular sheets, poorly defined trabeculae and pseudoglandular structure (×200 magnification). (b) Tumour cells showed mild atypias with nucleolar enlargement a slightly increased nuclear/cytoplasmatic ratio and nuclear crowding. No mitotic activity was observed but the proliferative rate was found slightly increased (×400 magnification).

A 54-year-old woman was for the first time admitted to our hospital in 1996 for evaluation of recurrent episodes of hypoglycemia associated with neuroglycopenic symptoms. To evaluate the repeated episodes of spontaneous hypoglycemia, she underwent the prolonged fast. Ten hours after the last ingestion of food, her plasma glucose was 1.8 mmol/L, serum insulin and C-peptide were 22.8 mE/L, and 0.97 nmol/L respectively, clearly indicating endogenous hyperinsulinism. Preoperatively, a tumor mass was localized at the head of the pancreas by endoscopic ultrasound and confirmed by a selective arteriography of the arteria mesenterica superior. Surgical exploration confirmed the preoperative localization and a well demarcated, roundish tumor with a diameter of 15–18 mm was completely enucleated. Histopathological examination confirmed an islet cell adenoma, and the tumor was positive for insulin by immunostaining (Figure 1(a)). Tumor cells showed mild atypias with nucleolar enlargement, a slightly increased nuclear/cytoplasmatic ratio and nuclear crowding. No mitotic activity was observed but the proliferative rate, measured by Ki-67 index of 5%, was found slightly increased. There were no histomorphological signs which might suggest malignancy (Figure 1(b)). Macroscopically, there were no signs of either invasion or metastases. After surgical excision of the tumor, the patient was relieved from hypoglycemic attacks. Postoperatively, her serum insulin and C-peptide level were in normal range. The patient was without any symptoms of the disease and without significant abnormalities in laboratory data for the next 14 years. 

**Figure 2:**
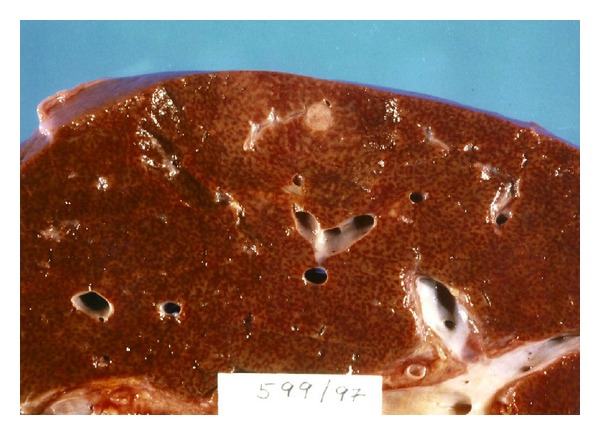
Gross appearance of liver in autopsy showed a few scattered, barely visible gray-whitish nodules with diameter varying from 2 to 6 mm. Light microscopy clearly revealed scattered minute roundish tumours, which were declared to be metastases.

The 69-year-old patient again experienced repeated episodes of seizure and conscious disturbance upon fasting. She was readmitted to our hospital. A fasting test again confirmed hypoglycemia and endogenous hyperinsulinism with a serum glucose values 1.6 mmol/L, serum insulin and C-peptide were 24.8 mE/L, and 1.03 nmol/L respectively. All currently available radiological imaging techniques could not confirm our suspicion for either recurrence of pancreatic insulinoma or metastatic growth. Repeated ultrasonography, MRI, and CT with contrast revealed scattered tiny lesions in the liver, as might be seen in cases of multiple hemangioma. Percutaneous needle biopsy of the liver and explorative laparotomy were refused by the patient (Figure 2). She was dismissed to home care with oral administration of diazoxide to prevent hypoglycemic attacks and instructions for a specific dietary regime. Six months later the patient was readmitted to our department of nephrology due to fully developed symptoms and laboratory signs of nephritic syndrome and rapidly progressing renal insufficiency. Two repeated percutaneous needle kidney biopsies revealed pauci-immune crescentic glomerulonephritis. Atypical P-ANCA was positive in low titer 1 : 80 by indirect immunofluorescence. Her hypoglycemic attacks got worse and became more persistent. The patient had to be treated with continuous infusions of 40% glucose. Later she developed thrombosis of the left iliofemoral vein and both sides jugular veins, accompanied with ileus, which was fatal for our patient.

## 3. Discussion

It is well established that it is difficult to predict the malignant nature of an insulinoma on the basis of its histological features [7, 8]. The current WHO classification criteria involve the presence of metastases, gross invasion, tumor size percentage of mitoses, proliferative index, and vascular invasion. However, metastases are generally considered the only definitive characteristic of malignancy. Out of all insulinomas, 5–10% are malignant [5, 6].

In our patient, the diagnosis of insulinoma was made clinically 15 years before the death by notifying characteristic Whipple's triad and a confirmed endogenous hyperinsulinism by a 72-hour supervised fast. The histomorphological study associated with initial surgery 15 years before the death of our patient suggested the diagnosis of a benign pancreatic endocrine tumor, compatible with a functioning insulin-producing adenoma. Reevaluation of the histomorphology and a recent application of immunohistochemistry have not only confirmed a pancreatic endocrine insulin producing tumor, but also enabled reclassification of the tumor into pancreatic insulinoma of uncertain malignant potential according to the criteria suggested by Solcia et al. [9]. The prediction of biological behavior of pancreatic endocrine tumors has been generally regarded as uncertain and the only unequivocal evidence of malignancy is gross invasion of adjacent organs, metastases to regional lymph nodes, liver and other distant sites, or histologically convincing angioinvasion [10, 11]. None of these finding was proven at initial surgery, nor in the pathological evaluation of the surgical specimen in our patient. Another case has been described in the literature, interesting due to some similarities related to the unusual course of the pancreatic endocrine neoplastic disease. Sata et al. [12] reported a rare case of malignant pancreatic insulinoma which recurred as a multiple metastasis 8 years after the initial complete enucleation of a primary tumor diagnosed originally, as in our patient as a benign adenoma with insulin production. Our case study demonstrates a similar recurrence after 15 years of full recovery following the initial surgery of an insulinoma with a fairly benign outlook and a similar size but with some tentative worse histomorphological parameters observed at the tumor periphery. Our study confirms the indolent and apparently extremely slow growing, but nevertheless malignant behavior that an insulinoma may show.

Furthermore, in our patient, an unusual, not yet described association of two diseases was established. An apparently extremely slowly growing metastasizing pancreatic insulinoma was found after 15 years of followup accompanied by ANCA positive crescentic glomerulonephritis. It may only be a coincidence of two causally unrelated diseases. However, a casual relationship between neoplastic diseases and immune complex forms of glomerulonephritis has been firmly established, and even cases of neoplastic diseases accompanied by pathogenetically undefined crescentic glomerulonephritis have occasionally been reported [13]. Our patient did not present a form of immune complex-mediated glomerulonephritis but had a crescentic glomerulonephritis which was ANCA associated. Incidental case reports have mentioned ANCA in neoplastic disorders, although their pathogenetic role remains obscure [13]. Furthermore, it has been reported that certain drugs such as thiouracils, hydralazine are capable of inducing ANCA positive systemic vasculitis, which resolved with discontinuation of the drug [14]. Our patient was given diazoxide due to recurrent hyperinsulinism. The association of diazoxide treatment and the occurrence of ANCA-associated glomerulonephritis or systemic vasculitis has not so far been described but in our opinion this possibility has to be considered.

## References

[1] Halfdanarson TR, Rubin J, Farnell MB, Grant CS, Petersen GM (2008). Pancreatic endocrine neoplasms: epidemiology and prognosis of pancreatic endocrine tumors. *Endocrine-Related Cancer*.

[2] Service FJ, Mcmahon MM, O’Brien PC, Ballard DJ (1991). Functioning insulinoma: a 60-year study. *Mayo Clinic Proceedings*.

[3] Vaidakis D, Karoubalis J, Pappa T, Piaditis G, Zografos GN (2010). Pancreatic insulinoma: current issues and trends. *Hepatobiliary and Pancreatic Diseases International*.

[4] Grant CS (2005). Insulinoma. *Best Practice and Research*.

[5] Kimura W, Kuroda A, Morioka Y (1991). Clinical pathology of endocrine tumors of the pancreas. *Digestive Diseases and Sciences*.

[6] Alexakis N, Neoptolemos JP (2008). Pancreatic neuroendocrine tumours. *Best Practice and Research in Clinical Gastroenterology*.

[7] Jensen RT (2006). Pancreatic neuroendocrine tumors: overview of recent advances and diagnosis. *Journal of Gastrointestinal Surgery*.

[8] Nikfarjam M, Warshaw AL, Axelrod L (2008). Improved contemporary surgical management of insulinomas: a 25-year experience at the massachusetts general hospital. *Annals of Surgery*.

[9] Solcia E, Capella C, Kloppel G (1997). Tumors of the pancreas. *Atlas of Tumor Pathology*.

[10] Kaltsas GA, Besser GM, Grossman AB (2004). The diagnosis and medical management of advanced neuroendocrine tumors. *Endocrine Reviews*.

[11] Mittendorf EA, Liu YC, Mchenry CR (2005). Giant insulinoma: case report and review of the literature. *Journal of Clinical Endocrinology and Metabolism*.

[12] Sata N, Kimura W, Kanazawa T, Muto T (1995). Malignant insulinoma causing liver metastasis 8 years after the initial surgery. *Surgery Today*.

[13] Burstein DM, Korbet SM, Schwartz MM (1993). Membranous glomerulonephritis and malignancy. *The American Journal of Kidney Diseases*.

[14] Couser WG (1982). Idiopathic rapidly progressive glomerulonephritis. *The American Journal of Nephrology*.

